# Epidemiological and clinical characteristics of imported malaria patients admitted to a specialized hospital: a retrospective study (2021– June 2026)

**DOI:** 10.3389/fcimb.2026.1952587

**Published:** 2026-09-21

**Authors:** Kun Peng, Wenjing Gong, Xuan Dong, Yunjing Zhao, Li Zhang, Chuanbing Zhu, Nuo Xu, Xiang Li, Yihan Guo, Yanmei Shen, Hongli Zhao

**Affiliations:** Digestive Disease Hospital of Shandong First Medical University, Shandong First Medical University & Shandong Academy of Medical Sciences, Jining, Shandong, China

**Keywords:** imported malaria, malaria elimination, *Plasmodium falciparum*, retrospective study, travel history

## Abstract

**Background:**

China was certified malaria-free by the World Health Organization in 2021; however, imported malaria remains a significant challenge to maintaining malaria elimination due to increasing international travel and overseas population mobility. Imported malaria cases have been continuously reported in Shandong Province, but detailed clinical characteristics, parasite species distribution, travel history, and treatment patterns among hospitalised malaria patients following elimination remain insufficiently characterised.

**Methods:**

We conducted a retrospective hospital-based observational study of malaria patients admitted to our hospital in Shandong Province, China, from January 2021 to June 2026. Demographic information, travel history, clinical manifestations, laboratory findings, *Plasmodium* species identification, and antimalarial treatments were analysed. The associations between travel regions and *Plasmodium* species distribution were evaluated using chi-square tests.

**Results:**

A total of 120 hospitalised malaria patients were included in the analysis. The number of malaria admissions increased from two cases in 2021 to 45 cases in 2025, with seasonal clustering during the second and third quarters of the year. All patients were male, and 98.3% were aged 30–59 years. Most patients had a travel history to Africa (95.8%), particularly West Africa (54.2%) and Central Africa (28.3%). Among the 120 patients with confirmed parasite identification, *Plasmodium falciparum* (*P. falciparum*) was the predominant species (67.5%), followed by *Plasmodium ovale* (*P. ovale*) (20.0%), *Plasmodium vivax* (*P. vivax*) (7.5%), and *Plasmodium malariae* (*P. malariae*) (5.0%). Fever was observed in all patients. The distribution of *Plasmodium* species differed significantly according to travel regions (Monte Carlo simulated *P* < 0.001). Further analysis within Africa revealed a significant difference among West, Central, and East Africa (Monte Carlo simulated *P* = 0.016). All patients received antimalarial treatment. Artemisinin-based therapies were the main treatment strategies, with dihydroartemisinin–piperaquine used in 90.0% of patients and intravenous artesunate administered in 41.7% of cases.

**Conclusion:**

Imported malaria remains a significant clinical and public health challenge following malaria elimination in Shandong Province. Cases are predominantly identified among working-age males returning from sub-Saharan Africa, with P. falciparum as the dominant parasite species. Therefore, strengthening surveillance of overseas travellers, improving early diagnosis, and maintaining standardised treatment protocols are essential to prevent the re-establishment of malaria.

## 1 Background

Malaria remains one of the most significant parasitic infectious diseases worldwide, posing a substantial threat to global public health. Despite considerable progress in malaria control and elimination, millions of malaria cases continue to occur annually, particularly in tropical and subtropical regions (Platon et al., 2026). *P. falciparum* infection accounts for the majority of malaria-related morbidity and mortality, due to its rapid progression, high parasite burden, and potential to cause severe complications (Petter and Duffy, 2015; Berners-Lee and Smith, 2025; Shah and Karmodiya, 2025).

In 2021, China was certified malaria-free by the World Health Organization (WHO) following decades of sustained control efforts (Zhu et al., 2024). However, imported malaria cases continue to occur due to increasing international travel, overseas employment and population mobility. Imported malaria, particularly from endemic regions in Africa, remains a major challenge to maintaining the malaria-free status (Lu et al., 2014; Lai et al., 2019; Talapko et al., 2019; Liu et al., 2021a; Kamana and Zhao, 2023).

Shandong Province, located in eastern China, achieved malaria elimination through the national elimination programme. However, as a province with substantial international economic activity and a large population engaged in overseas employment, imported malaria cases have been continually reported in Shandong. Previous investigations have shown that imported malaria cases in Shandong were predominantly acquired in African countries, with *P. falciparum* being the most common species (Xu et al., 2016; Yu et al., 2020; Shi et al., 2024).

Although previous studies have described the epidemiological characteristics of imported malaria at the provincial level, detailed clinical features, laboratory abnormalities, parasite species distribution, travel histories, and treatment patterns among hospitalised malaria patients following malaria elimination remain insufficiently characterised. Hospital-based clinical data are especially valuable for understanding disease severity, identifying early warning signs, and optimising clinical management strategies.

Therefore, this retrospective study aimed to characterise the epidemiological, clinical, laboratory, and therapeutic features of malaria patients admitted to our hospital in Shandong Province from January 2021 to June 2026. The findings provide updated evidence regarding patterns of imported malaria following malaria elimination and may contribute to improving clinical diagnosis, treatment, and surveillance strategies.

## 2 Methods

### 2.1.Study design and study population

This retrospective hospital-based observational study was conducted at our hospital in Shandong Province, China. We reviewed the medical records of all patients diagnosed with malaria and admitted between January 2021 and June 2026.

Patients were eligible for inclusion if they met the following criteria: (1) malaria infection confirmed by laboratory examination, including microscopic analysis of peripheral blood smears, rapid diagnostic tests (RDTs), or molecular detection; (2) admission to our hospital during the study period; and (3) availability of complete epidemiological and clinical information. Patients without laboratory confirmation, duplicate records, or incomplete clinical data were excluded. Of the 124 cases, one duplicate and three cases in which the malaria parasite species could not be identified were excluded; ultimately, 120 malaria patients were included in the analysis. Ethical approval was granted by the Medical Ethics Committee of the Digestive Diseases Hospital of Shandong First Medical University. This retrospective observational clinical study was exempt from the informed consent procedure; the approval number is 202302.

### 2.2 Epidemiological data collection

Demographic and epidemiological information was extracted from electronic medical records and malaria surveillance databases. The variables collected included age, sex, date of admission, travel history, countries visited prior to illness onset, and potential exposure regions. Travel destinations were categorised according to geographic regions, including West Africa, Central Africa, East Africa, Asia, and Oceania.

### 2.3 Clinical assessment and treatment

Clinical characteristics were recorded at admission, including fever, chills, headache, fatigue, myalgia, gastrointestinal symptoms, and other malaria-related manifestations. Malaria diagnosis was confirmed by microscopic identification of parasites in peripheral blood smears and/or molecular detection. The infecting *Plasmodium* species were classified as *P. falciparum*, *P. ovale*, *P. vivax*, and *P. malariae*. Mixed infections were noted when more than one parasite species was detected.

Antimalarial treatment information was obtained from medical records, including drug type, route of administration, treatment duration, and supportive therapy. We classified disease severity in cases in accordance with the WHO severity classification document (Organization, 2022).

### 2.4 Statistical analysis

For categorical data, variables were summarised as frequencies and percentages. The associations between travel regions and *Plasmodium* species distribution were assessed using cross-tabulation with chi-square tests. When the assumptions of the chi-square test were violated (i.e., more than 20% of cells had expected frequencies below five), the Monte Carlo approximation of Fisher’s exact test (10,000 samples, 99% confidence interval) was employed to obtain reliable p-values.

For continuous data: As most laboratory parameters were not normally distributed, continuous variables are presented as medians with interquartile ranges (IQR). Comparisons between two independent groups (e.g., severe versus non-severe *P. falciparum* cases) were performed using the Mann–Whitney U test. For the seven independent comparisons made between severe and non-severe groups, the significance level was adjusted using the Bonferroni method to α′ = 0.00714 (0.05/7) to control for Type I error. Comparisons among multiple groups (e.g., across *Plasmodium* species) were performed using the Kruskal–Wallis H test. When a statistically significant overall difference was detected, *post hoc* pairwise comparisons were conducted with Bonferroni correction for multiple testing.

For all analyses, a two-sided P value < 0.05 was considered statistically significant, unless otherwise specified. When the total sample size for a subgroup comparison was ≤30, exact significance values were reported instead of asymptotic approximations.

## 3 Results

### 3.1 Temporal distribution of hospitalized malaria cases

From January 2021 to June 2026, a total of 120 malaria patients (**Table 1**) were admitted to our hospital in Shandong Province, China. As the cases for 2026 only cover the period from January to June, including them would introduce bias into the analysis in this section; therefore, the 17 cases from 2026 have been excluded from this subsection of the study. The annual number of admitted malaria patients showed an increasing trend during the study period, rising from two cases in 2021 to 45 cases in 2025. Cases occurred throughout the year, with clear seasonal variation. The highest number of cases was observed in August (14/103, 13.6%), followed by September (13/103, 12.6%) (**Figure 1**). Quarterly analysis showed that the majority of cases occurred during the second and third quarters (65/103, 63.1%), with the third quarter accounting for the largest proportion (39/103, 37.9%).

**Table 1:** Temporal distribution of hospitalized malaria cases in Shandong Province, China, January 2021–June 2026.

Year	Jan	Feb	Mar	Apr	May	Jun	Jul	Aug	Sep	Oct	Nov	Dec	Total
2021	0	0	0	0	1	0	1	0	0	0	0	0	2
2022	1	1	0	0	0	0	0	1	0	0	0	0	3
2023	0	2	0	3	4	3	4	4	1	2	0	3	26
2024	4	1	2	0	2	2	2	4	5	0	4	1	27
2025	4	1	3	3	2	6	5	5	7	2	2	5	45
2026*	4	2	1	2	4	4	–	–	–	–	–	–	17
Total	**13**	**7**	**6**	**8**	**13**	**15**	**12**	**14**	**13**	**4**	**6**	**9**	**120**

*Data available until June 2026. The figures in bold represent the sum of the values in each column.

**Figures 1:**
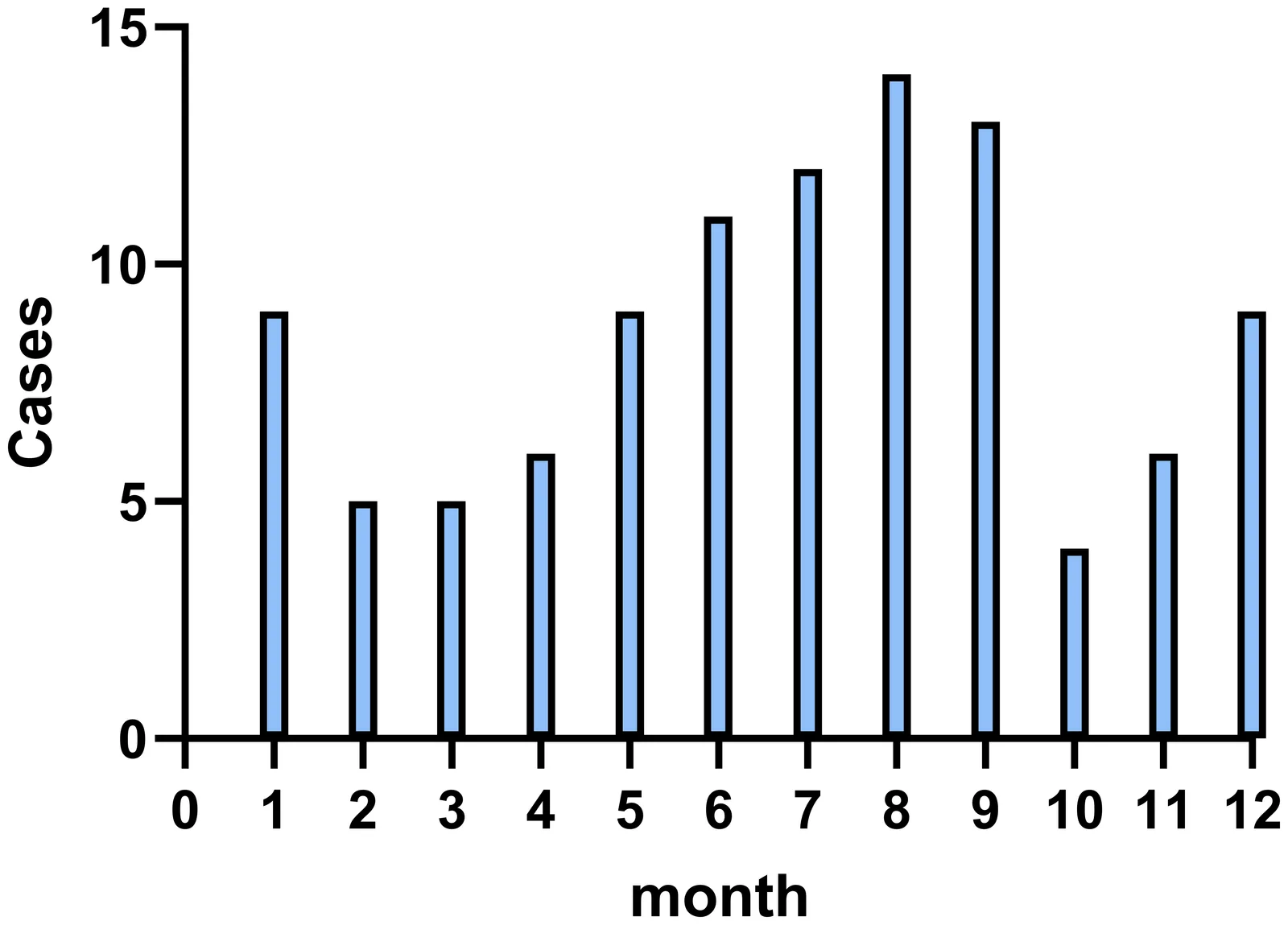
Monthly distribution of malaria cases .

### 3.2 Demographic characteristics of hospitalized malaria patients

All patients were male (120/120, 100.0%) (**Table 2**) during the study period. The patients were predominantly middle-aged adults. The largest proportion was observed in the 30–39-year age group (49/120, 40.8%), followed by the 40–49-year age group (39/120, 32.5%) and the 50–59-year age group (30/120, 25.0%). Only one patient was aged 20–29 years (0.8%) and one patient was aged ≥60 years (0.8%), while no patients younger than 20 years were recorded. Overall, patients aged 30–59 years accounted for 98.3% (118/120) of all hospitalised malaria cases, indicating that malaria admissions to our hospital mainly occurred among working-age adult males.

**Table 2:** Demographic characteristics of malaria patients admitted to our hospital during 2021–2026.

Characteristics	No. of patients (%)
Sex	
Male	120 (100.0)
Female	0 (0.0)
Age group (years)	
0–9	0 (0.0)
10–19	0 (0.0)
20–29	1 (0.8)
30–39	49 (40.8)
40–49	39 (32.5)
50–59	30 (25.0)
≥60	1 (0.8)

### 3.3 Travel history and geographic origin of malaria patients

Among the 120 malaria patients admitted to our hospital from January 2021 to June 2026, travel histories indicated that the majority had recent exposure in Africa (115/120, 95.8%), while only a small proportion reported travel to Asia (3/120, 2.5%) and Oceania (2/120, 1.7%). West Africa was the predominant region of exposure, accounting for 65 cases (54.2%), followed by Central Africa (34 cases, 28.3%) and East Africa (16 cases, 13.3%). Guinea was the most frequently reported country of exposure (31 cases, 25.8%), followed by the Democratic Republic of the Congo and Nigeria (20 cases, 16.7%) (**Table 3**). Overall, imported malaria cases in our hospital were mainly associated with travel to sub-Saharan African countries, particularly West and Central Africa, reflecting the major geographic distribution of malaria exposure among imported cases in Shandong Province.

**Table 3:** Geographic distribution of travel history among malaria patients admitted to our hospital, Shandong Province, China, January 2021–June 2026.

West Africa (n=65)	Central Africa (n=34)	East Africa (n=16)	Asia (n=3)	Oceania (n=2)
Nigeria (n=20)	Chad (n=1)	Ethiopia (n=2)	Indonesia (n=1) Pakistan (n=2)	Solomon Islands (n=1) Papua New Guinea (n=1)
Liberia (n=2)	Cameroon(n=4)	Uganda (n=4)		
Côte d'Ivoire (n=9)	Gabon (n=3)	Tanzania (n=2)		
Ghana (n=1)	Congo (DRC) (n=20)	Zambia (n=3)		
Guinea (n=31)	Republic of the Congo (n=3)	Mozambique(n=3)		
Burkina Faso(n=1)	Equatorial Guinea (n=1)	Republic of Kenya (n=1)		
Senegal (n=1)	Angola (n=2)	Madagascar(n=1)		

### 3.4 Distribution of *Plasmodium* species and clinical manifestations

Among the 120 malaria patients admitted to our hospital, *Plasmodium* species identification was successfully performed in all cases. *P. falciparum* was the predominant species, accounting for 81 cases (67.5%) (**Figure 2A**), followed by *P. ovale* (24 cases, 20.0%) (**Figure 2B**), *P. vivax* (9 cases, 7.5%) (**Figure 2C**), and *P. malariae* (6 cases, 5.0%) (**Figure 2D**), No mixed infections were detected among the identified cases.

**Figures 2:**
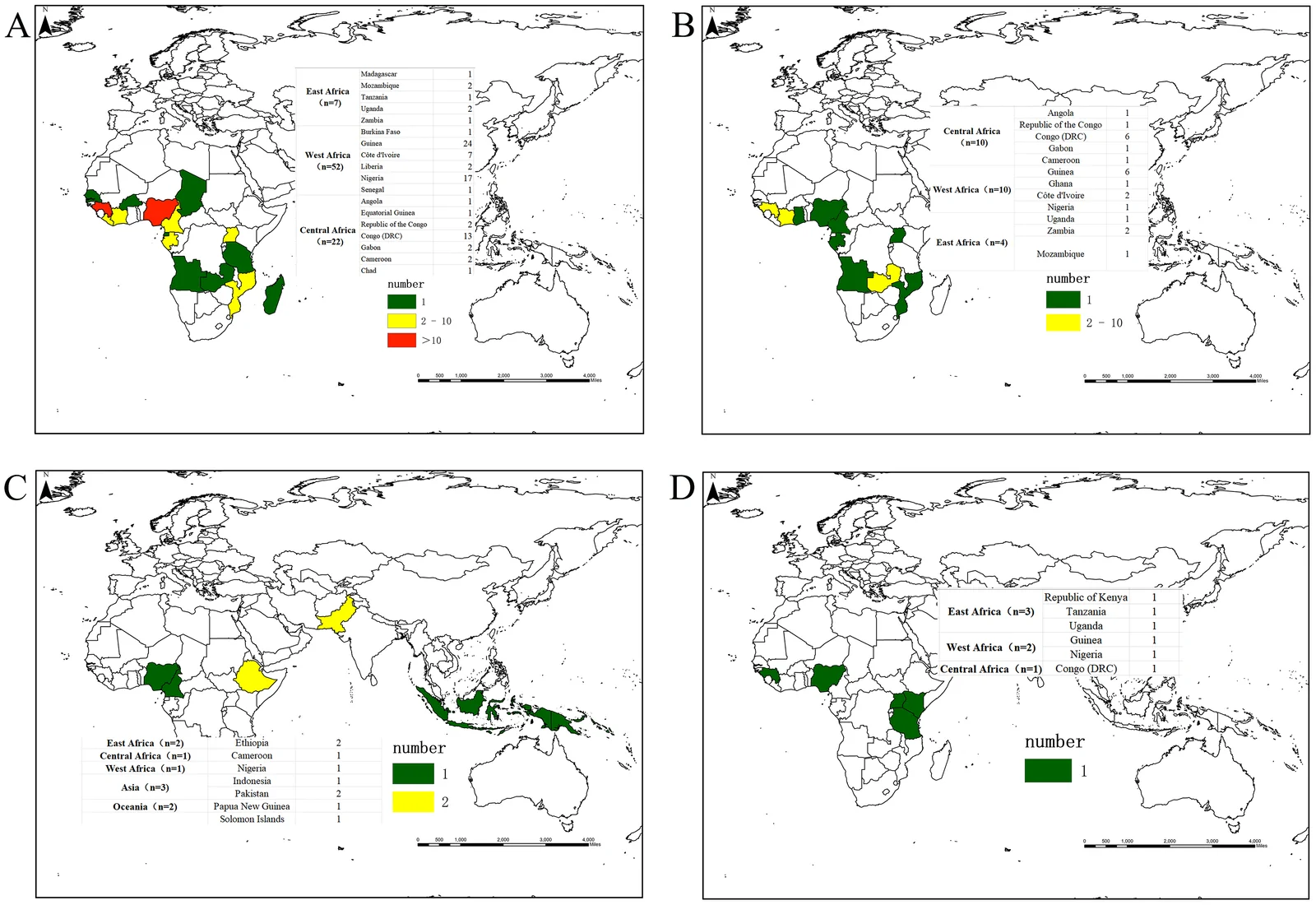
Travel history of patients with malaria **(A)** *P. falciparum*. The cases spanned the period from 2023 to 2026 and originated in West, Central, and East Africa. **(B)** *P. ovale*. The cases span the period from 2021 to 2026 and originate from West, Central, and East Africa. **(C)** *P. vivax*. The cases span the period from 2022 to 2025 and originate from West Africa, Central Africa, East Africa, Asia, and Oceania. **(D)** *P. malariae*. The cases spanned the period from 2023 to 2026 and originated in West, Central, and East Africa.

Fever was observed in all identified malaria patients (120/120, 100.0%), making it the most common clinical manifestation. A proportion of patients exhibited laboratory abnormalities or clinical features associated with severe malaria, including thrombocytopenia, liver dysfunction, and a high parasite burden.

### 3.5 Association between travel history and *Plasmodium* species distribution

The distribution of *Plasmodium* species varied significantly according to the geographic regions of travel history (Pearson *χ²* = 64.3, *df* = 6, Monte Carlo simulated *P* < 0.001) (**Table 4**). *Post hoc* pairwise comparisons using Bonferroni correction (α′ = 0.0167) revealed that the species distribution in Africa differed significantly from that in Asia (Monte Carlo simulation *P* < 0.001) and Oceania (Monte Carlo simulation *P* = 0.004), whereas no significant difference was observed between Asia and Oceania (all cases in these two regions were infections with *P. vivax*; Monte Carlo P-value = 1.000) (**Supplementary Table S1**). *P. falciparum* was the predominant species among patients returning from African endemic regions, particularly West Africa, where it accounted for 80.0% (52/65) of infections. In Central Africa, although *P. falciparum* remained dominant at 64.7% (22/34), the proportion of *P. ovale* infections was relatively higher at 29.4% (10/34) compared with other regions. East Africa exhibited a more diverse parasite composition, including *P. falciparum*, *P. ovale*, *P. vivax*, and *P. malariae*. All cases originating from Asia were identified as *P. vivax* infections.

**Table 4:** Distribution of *Plasmodium* species according to travel regions among imported malaria patients.

Plasmodium species	West Africa (n=65)	Central Africa (n=34)	East Africa (n=16)	Africa (n=115)	Asia (n=3)	Oceania (n=2)	Total (n=120)
*P. falciparum*	52(80.0%)	22(64.7%)	7 (43.8%)	81 (70.4%)	0 (0.0%)	0 (0.0%)	81 (67.5%)
*P. ovale*	10(15.4%)	10(29.4%)	4 (25.0%)	24 (20.9%)	0 (0.0%)	0 (0.0%)	24 (20.0%)
*P. vivax*	1 (1.5%)	1 (2.9%)	2 (12.5%)	4 (3.5%)	3 (100.0%)	2 (100.0%)	9 (7.5%)
*P. malariae*	2 (3.1%)	1 (2.9%)	3 (18.8%)	6 (5.2%)	0 (0.0%)	0 (0.0%)	6 (5.0%)
Total	65 (100%)	34 (100%)	16 (100%)	115 (100%)	3 (100%)	2 (100%)	120 (100%)

Monte Carlo simulation yielded *P* < 0.001 for the comparison of species distribution across Africa, Asia, and Oceania.

Monte Carlo simulation yielded a P-value of 0.016 for the comparison of species distribution among West, Central, and East Africa (African sub-regional analysis). The effect size for this analysis, measured by Cramér’s V, was 0.263.

Due to sparse data (more than 20% of cells had expected frequencies below 5 in both analyses), the Monte Carlo approximation of Fisher’s exact test (10,000 samples, 99% confidence interval) was applied to ensure valid statistical inference.

The percentages in brackets represent the proportion within each row; as they are rounded to one decimal place, the total of these percentages is approximately 100%.

### 3.6 Association between African travel regions and *Plasmodium* species distribution

Among the 115 malaria patients with travel histories to African countries, West Africa was the most common region of exposure (65/115, 56.5%), followed by Central Africa (34/115, 29.6%) and East Africa (16/115, 13.9%). *P. falciparum* was the predominant parasite species in all African regions, accounting for 80.0% (52/65), 64.7% (22/34), and 43.8% (7/16) of infections in West, Central, and East Africa, respectively. *Plasmodium* species differed significantly among West, Central, and East Africa (Pearson *χ²* = 15.877, *df* = 6, Monte Carlo simulated *P* = 0.016, 99% CI: 0.013–0.019) (**Table 4**). The effect size, measured by Cramér’s V, was 0.263, indicating a small to moderate association between travel region and parasite species. *Post hoc* pairwise comparisons with Bonferroni correction (α′ = 0.0167) revealed that the significant overall difference among African sub-regions was primarily driven by the contrast between West and East Africa (Monte Carlo simulated *P* = 0.014). No significant differences were observed between West and Central Africa (Monte Carlo simulated *P* = 0.253) or between Central and East Africa (Monte Carlo simulated *P* = 0.115) (**Supplementary Table S1**).

### 3.7 Clinical characteristics and laboratory parameters

Among the 81 patients with *P. falciparum*, 23 (28.4%) were classified as having severe malaria according to WHO criteria. As shown in **Table 5**, compared with non-severe cases, patients with severe malaria had significantly higher median parasite densities [67,040 (IQR: 20,460–238,500)/µL versus 6,030 (IQR: 3,068.5–17,051.3)/µL], elevated lactate dehydrogenase (LDH) levels [497.2 (IQR: 356.2–709.0) U/L versus 318.4 (IQR: 253.6–401.5) U/L], and longer hospital stays [7 (IQR: 6–9.8) days versus 5.5 (IQR: 4–7) days] (all *P* < 0.001, Mann–Whitney U test). After Bonferroni correction for multiple comparisons (significance level set at α′ = 0.00714), parasite density, LDH, and length of stay remained significantly different between the two groups. Although haemoglobin levels tended to be lower and ALT levels higher in severe cases, these differences did not reach statistical significance after correction (*P* = 0.018 and *P* = 0.045, respectively).

**Table 5:** Differences in clinical indicators between the severe and non-severe *P. falciparum* malaria groups.

Indicators	Severe group median (P25, P75)/N	Non-severe group median (P25, P75)/N	P	Significance after correction*
Density (/µL)	67040 (20460-238500)/(n=19)	6030 (3068.5-17051.3)/(n=40)	<0.001	*
Hb (g/L)	89.5 (76.5-114.5)/(n=8)	140 (105-155.8)/(n=20)	=0.018	
PLT (×10^9^/L)	44 (35-90)/(n=23)	72 (58-91)/(n=31)	=0.16	
ALT (U/L)	103.7 (77.1-194.5)/(n=10)	66.7 (54.8-90.1)/(n=19)	=0.045	
GGT (U/L)	108.9 (71.1-160.7)/(n=14)	98.7 (79.5-128.6)/(n=27)	=0.441	
LDH (U/L)	497.2 (356.2-709.0)/(n=22)	318.4 (253.6-401.5)/(n=40)	<0.001	*
Length of stay (d)	7 (6-9.8)/(n=20)	5.5 (4-7)/(n=54)	<0.001	*

The table presents the median and the interquartile range (IQR). When the total sample size exceeds 30, the p-value is based on asymptotic significance (two-tailed); when it is 30 or fewer, the p-value is based on exact significance.

As detection rates varied across different parameters, each analysis included only patients with malignant malaria for whom complete data were available for the specific parameter. The Mann–Whitney U test was used to compare the two groups. To control for Type I errors arising from multiple comparisons, the Bonferroni correction was applied, with the adjusted significance level set at α = 0.00714.

Comparison of laboratory parameters across different *Plasmodium* species revealed that parasite density differed significantly among species (*P* < 0.001, Kruskal–Wallis H test; **Table 6**). *Post hoc* pairwise comparisons with Bonferroni correction showed that *P. falciparum* infections had significantly higher parasite densities than both *P. ovale* (adjusted *P* = 0.001) and *P. malariae* (adjusted *P* = 0.004). No significant differences were observed among species for haemoglobin, platelet count, GGT, or LDH levels (all *P* > 0.05). Total bilirubin showed marginal differences among species (*P* = 0.01), but *post hoc* comparisons did not reach statistical significance after Bonferroni correction (**Supplementary Tables S2**, **S3**).

**Table 6:** Differences in various indicators between groups of different species of malaria parasites.

Indicators	*P. falciparum*	*P. ovale*	*P. vivax*	*P. malariae*	*P*
Density (/µL)	11500 (3750-57000)/(n=59)	1320 (612-3335)/(n=17)	4300 (2410-8620)/(n=7)	1000 (260-1680)/(n=5)	<0.001
Hb (g/L)	121.5 (94.3-154.5)/(n=28)	108 (99-143)/(n=7)	n=1	n=2	0.421
PLT (×10^9^/L)	61.5 (41.8-91)/(n=28)	67 (46.8-85)/(n=28)	55 (31-83)/(n=28)	n=3	0.283
GGT (U/L)	103.7 (79-134.4)/(n=41)	94.3 (70.8-118.8)/(n=8)	65.7 (64.4-130.9)/(n=4)	n=2	0.273
LDH (U/L)	347.3 (293.7-535.5)/(n=62)	398.1 (285.4-432.1)/(n=16)	254.7 (244.7-303.2)/(n=9)	325.6 (278.4-430.3)/(n=6)	0.078
TBIL (µmol/L)	34.2 (25.2-45.2)/(n=36)	27.0 (24.0-29.5)/(n=11)	22.2 (21.6-22.7)/(n=4)	n=0	0.01
DBIL (µmol/L)	14.0 (10.1-18.0)/(n=36)	10.6 (9.3-11.7)/(n=11)	9.5 (9.2-9.9)/(n=4)	n=0	0.051

A Kruskal-Wallis H test was conducted to compare the groups; however, for the ‘Hb’ parameter, as there were only two groups, a Mann-Whitney U test was performed on these two groups alone. Due to the small sample size for some parameters in the P. malariae group, the interquartile range was not calculated.

### 3.8 Antimalarial treatment and clinical management

All 120 malaria patients admitted to our hospital received antimalarial treatment. Artemisinin-based therapies were the primary treatment strategy. Dihydroartemisinin–piperaquine was the most frequently administered regimen, used in 108 patients (90.0%). Primaquine was added for radical cure in 30 patients (25.0%), according to parasite species and clinical requirements.

Intravenous artesunate-containing regimens were administered to 50 patients (41.7%), primarily those with severe clinical manifestations, high parasite density, or requiring rapid parasite clearance. Following clinical improvement, most patients were switched to oral artemisinin-based combination therapy to complete their treatment.

Of the 120 patients, five did not complete their treatment due to a strong desire to be discharged; 42 were discharged after their condition improved, and 73 were discharged following a full recovery.

## 4 Discussion

In this hospital-based retrospective study, we analysed the epidemiological and clinical characteristics of 120 malaria patients admitted to our hospital in Shandong Province from January 2021 to June 2026. The number of hospitalised cases increased from two in 2021 to 45 in 2025. This rise corresponds with the lifting of China’s strict COVID-19 control policies on 8 January 2023, when international travel restrictions were removed (Zeyu et al., 2024). Nationally, reported malaria cases surged from 799 in 2021 to 4,314 in 2025, with a 194.4% increase in 2023 alone, reflecting our local trend. Shandong Province ranked fourth nationally in 2023 (178 cases) and 2024 (232 cases), and fifth in 2025 (307 cases) (Zhang and Xia, 2024; Zhang et al., 2025a; Zhang et al., 2026). Most cases in our study occurred during the second and third quarters, consistent with national seasonal patterns (Shi et al., 2017; Sun et al., 2023; Wu et al., 2023; An et al., 2024).

A notable finding of our study was the complete predominance of male patients, with 120/120 (100.0%) occurring in men, and 98.3% of patients aged 30–59 years. This demographic pattern is consistent with previous reports from China, where imported malaria cases are predominantly observed among working-age males (Kong et al., 2017; Liu et al., 2021b; Liang et al., 2022). The predominance of this population is likely linked to overseas labour migration, particularly in construction, mining, engineering, and commercial activities within African malaria-endemic countries. These findings underscore the importance of enhancing malaria prevention education, personal protection measures, and screening strategies for overseas workers both prior to and following international travel.

The geographic distribution of infection sources demonstrated that imported malaria cases in our hospital were overwhelmingly associated with sub-Saharan Africa (95.8%).West Africa was the most common region of exposure, followed by Central and East Africa, which is consistent with national surveillance data indicating that Africa has been the primary source of imported malaria cases in China during the post-elimination period (Lai et al., 2016; She et al., 2020; Zhao et al., 2020; Kamana et al., 2023). The strong association with African countries likely reflects both the high intensity of malaria transmission in sub-Saharan Africa and the extensive economic cooperation and population exchange between China and these countries.

Regarding parasite species, *P. falciparum* was the dominant species (67.5%), followed by *P. ovale* (20.0%), *P. vivax* (7.5%), and *P. malariae* (5.0%). The predominance of *P. falciparum* is consistent with previous studies of imported malaria in China, where it accounted for the largest proportion of imported infections, particularly among travellers returning from Africa (Feng et al., 2019; Xia et al., 2020; Xu et al., 2023; Zeng et al., 2025). The relatively high proportion of *P. ovale* cases (20%) observed in this study is not an isolated occurrence in China; a retrospective analysis of imported malaria cases in five Chinese provinces between 2014 and 2021 also reported a high detection rate of *P. ovale* (19%) (Liu et al., 2024). This elevated proportion may be attributable to the fact that all patients in this study were migrant workers who had spent extended periods in malaria-endemic regions of Africa, where they were repeatedly exposed to a high risk of malaria infection, thereby increasing their likelihood of *P. ovale* infection. This finding is consistent with a study conducted in Anhui Province (Zhang et al., 2025b). We further evaluated the relationship between travel regions and parasite species. Although *P. falciparum* was the predominant species across all three African sub-regions, its proportion varied significantly, decreasing from 80.0% in West Africa to 64.7% in Central Africa and 43.8% in East Africa (Monte Carlo simulated *P* = 0.016). Regional trends included a higher proportion of *P. ovale* in Central Africa (29.4%) and a more diverse parasite composition in East Africa. The detailed travel history remains essential for guiding laboratory diagnosis and clinical management, as parasite distribution varies geographically.

All patients with confirmed malaria presented with fever, emphasising that unexplained fever in returning travellers should prompt consideration of a malaria diagnosis. Previous studies have demonstrated that delayed diagnosis and treatment are significant risk factors for severe imported malaria, particularly in cases of *P. falciparum* infection (Gong and Wan, 2025). Therefore, clinicians should maintain a high index of suspicion for malaria in patients with travel histories to endemic regions, even after local malaria elimination has been achieved. In patients with a single infection of malignant malaria, those with severe disease exhibited higher parasite densities, significantly elevated lactate dehydrogenase (LDH) levels, and significantly longer hospital stays compared with non-severe cases. LDH, as a marker of cellular damage and haemolysis, is particularly elevated in severe cases, suggesting that routine LDH testing can serve as a rapid and cost-effective adjunctive indicator for assessing disease severity in clinical settings where quantitative parasite density cannot be immediately determined. When comparing laboratory parameters across the four malaria parasite species, only parasite density showed a significant difference, with *P. falciparum* having the highest median density (11,500/µL), followed by *P. vivax* (4,300/µL), *P. ovale* (1,320/µL) and *P. malariae* (1,000/µL) (*P* < 0.001). This gradient is consistent with the known biological characteristics of each species: *P. falciparum* can invade all stages of red blood cells, thereby producing higher parasitaemia, whilst *P. ovale* and *P. vivax* prefer young or ageing red blood cells, respectively, resulting in lower peripheral blood parasite densities (McKenzie and Bossert, 1999). It is worth noting that, despite differences in density, after correction for multiple comparisons, there were no significant differences in haemoglobin, platelet count, GGT, and LDH levels between the different species. This suggests that, although *P. falciparum* infection is associated with a higher parasite burden, the extent of haematological and hepatic abnormalities among hospitalised patients may be similar across the different *Plasmodium* species. This further emphasises the importance of species identification in guiding radical treatment (e.g. the use of primaquine for *P. vivax* and *P. ovale*). Furthermore, it should be noted that when patients were surveyed in this study, none had any documented record of using prophylactic medication whilst travelling, reflecting a generally low level of awareness regarding malaria prevention among migrant workers. This finding suggests that strengthening pre-travel health education and guidance on prophylactic medication for high-risk groups is a critical aspect of malaria prevention and control efforts that urgently requires attention.

In terms of treatment, patients in our study primarily received artemisinin-based therapies, including dihydroartemisinin–piperaquine and intravenous artesunate for those with severe manifestations or a high parasite burden (Zhang et al., 2017; Zhao et al., 2021; Kong et al., 2022). These treatment strategies align with WHO recommendations for the management of uncomplicated and severe malaria. The clinical outcomes for patients in this study were generally favourable: 73 patients (60.8%) were discharged following full recovery, and 42 (35.0%) were discharged after showing improvement. No deaths or cases of permanent sequelae were recorded, and no patients required intensive care. These favourable outcomes may reflect the timely administration of artemisinin-based medicines and the availability of specialist malaria diagnosis and treatment at our centre. However, five patients (4.2%) terminated their hospitalisation prematurely for personal reasons and discharged themselves. This subgroup represents a potential public health concern, as incomplete treatment may lead to relapse and delayed parasite clearance. Health education and patient counselling regarding the importance of completing the full course of antimalarial treatment should be strengthened, particularly for patients with high parasite density or severe symptoms, to minimise the risk of poor treatment outcomes in the post-elimination era.

The core mechanism for malaria elimination and post-elimination surveillance in China is the “1-3-7” operational protocol, which stipulates: “1” – healthcare facilities must report cases within one day; “3” – disease control authorities must complete case verification and epidemiological investigations within three days; and “7” – investigations and containment measures at the outbreak site must be completed within seven days (Huang et al., 2022). The challenges facing Shandong Province following malaria elimination share similarities with those encountered by countries that have already eliminated the disease, such as Sri Lanka and El Salvador. These risks arise from the continued influx of imported cases and the persistent presence of local malaria vectors. The lessons for Shandong from these international experiences lie in establishing sensitive surveillance and rapid response mechanisms (for example, Sri Lanka has developed a surveillance system based on passive case detection (PCD), reactive case detection (RACD) and active case detection (PACD) (Mendis et al., 2024); while El Salvador has implemented the ‘Alerta SIS’ digital early-warning system, integrated into the national electronic health record, as an effective means of preventing the re-emergence of malaria in the post-elimination phase (Bank, 2026).

This study has several limitations. Firstly, as a retrospective study, our findings may not fully represent all imported malaria cases in Shandong Province. Cases diagnosed and treated at other healthcare institutions were not included, thereby introducing selection bias. Furthermore, as cases of pre-admission death were excluded, the possibility of survival bias should be considered. Secondly, due to the retrospective design, some laboratory parameters and clinical information were incomplete. Thirdly, recall and reporting bias regarding travel history cannot be ruled out, as travel information was obtained through patient interviews. Fourthly, although molecular surveillance for markers of antimalarial drug resistance has not been incorporated, it remains of paramount importance. The emergence of partial artemisinin resistance in the Greater Mekong Subregion and parts of Africa poses a potential threat (Tun et al., 2015; Amaratunga et al., 2019). In Shandong, where local cases originate primarily from Africa, establishing a surveillance mechanism for pfkelch13 mutations would provide an early warning of reduced treatment efficacy; this is particularly important given that 90.0% of patients in this study received dihydroartemisinin–piperaquine treatment, while 41.7% received intravenous artemether treatment. To overcome these limitations, future studies employing prospective and standardised data collection are required.

## 5 Conclusion

In conclusion, imported malaria remains a significant challenge following the elimination of malaria in China. In our hospital, cases were predominantly observed among working-age males returning from sub-Saharan Africa, with P. falciparum as the primary parasite species. Strengthening surveillance of overseas travellers, improving early diagnosis, and maintaining standardised treatment protocols are essential to prevent the re-establishment of malaria.

## Data Availability

The raw data supporting the conclusions of this article will be made available by the authors, without undue reservation.
