# Supplementary material for: Epidemiological and clinical characteristics of imported malaria patients admitted to a specialized hospital: a retrospective study (2021– June 2026)
Source: Front Cell Infect Microbiol. 2026 Sep 21;16:1952587. doi: 10.3389/fcimb.2026.1952587 (PMC13634818; doi:10.3389/fcimb.2026.1952587)
Supplement: Supplementary file 1 [file Table1.doc]

Table S1 Post-hoc pairwise comparisons of *Plasmodium* species distribution by travel region

| **Intercontinental Comparison** | **Monte Carlo P** | **Significance**  **after correction*（α′=0.0167)** |
| --- | --- | --- |
| Africa and Asia | ＜0.001 | * |
| Africa and Oceania | 0.004 | * |
| Asia and Oceania |  |  |
| **Within Africa** | | |
| West Africa and Central Africa | 0.253 |  |
| West Africa and East Africa | 0.014 | * |
| Central Africa and East Africa | 0.115 |  |

For a precise Monte Carlo test, as three pairwise comparisons are required for each analysis, the significance level must be corrected using the Bonferroni method to α' = 0.05/3 = 0.0167. A difference is considered significant only if P < 0.0167.

**Table S2 Pairwise comparisons of “Density”**

| **Indicators** | **Test Statistic** | **Standard Error** | **Standardized Test Statistic** | ***P*** | **Adjusted *P**** |
| --- | --- | --- | --- | --- | --- |
| *P. falciparum-P. ovale* | 27.454 | 7.032 | 3.904 | .000 | .001* |
| *P. falciparum-P. vivax* | 11.269 | 10.213 | 1.103 | .270 | 1.000 |
| *P. falciparum-P. malariae* | 40.183 | 11.899 | 3.377 | .001 | .004* |
| *P. Ovale-P. vivax* | -16.185 | 11.473 | -1.411 | .158 | .950 |
| *P. Ovale-P. malariae* | 12.729 | 12.997 | .979 | .327 | 1.000 |
| *P. Vivax-P. malariae* | 28.914 | 14.959 | 1.933 | .053 | .319 |

Bonferroni correction was applied for multiple comparisons. The adjusted P values were automatically generated by SPSS software.

**Table S3 Pairwise comparisons of “TBIL”**

| **Indicators** | **Test Statistic** | **Standard Error** | **Standardized Test Statistic** | ***P*** | **Adjusted *P*** |
| --- | --- | --- | --- | --- | --- |
| *P. falciparum-P. ovale* | 7.333 | 5.122 | 1.432 | 0.152 | 0.457 |
| *P. falciparum-P. vivax* | 22.333 | 7.835 | 2.850 | 0.004 | 0.013 |
| *P. Ovale-P. malariae* | 15 | 8.680 | 1.728 | 0.084 | 0.252 |

Bonferroni correction was applied for multiple comparisons. The adjusted P values were automatically generated by SPSS software.
